# Citric Acid‐Crosslinked Highly Porous Cellulose Nanofiber Foam Prepared by an Environment‐Friendly and Simple Process

**DOI:** 10.1002/gch2.202200090

**Published:** 2022-09-02

**Authors:** Dinesh, Hanbin Wang, Jaehwan Kim

**Affiliations:** ^1^ Creative Research Center for Nanocellulose Future Composites Department of Mechanical Engineering Inha University 100 Inha‐ro, Michuhol‐gu Incheon 22212 South Korea

**Keywords:** antioxidant activity, cellulose nanofibers, citric acid, highly porous foams, hydrophobicity, sound absorption

## Abstract

In this study, cellulose nanofiber (CNF) foams are prepared by an environment‐friendly, time‐saving, and simple process using bio‐based citric acid (CA) as a green crosslinking agent. Scanning electron microscope and Fourier transform infrared spectroscopy examine the foam morphology and confirm the crosslinking. The prepared foam shows a very high porosity (>98%) with a low density (24.02 mg cm^−3^) with more than 200% improvement in mechanical strength and modulus compared to the neat CNF foam. In addition, the inclusion of CA into CNF improves thermal stability, antioxidant activity, and hydrophobicity. Furthermore, the prepared foam demonstrates a good sound absorption behavior, suitable for environment‐friendly and lightweight sound‐absorbing foam.

## Graphical abstract

Using citric acid, a biobased green crosslinking agent, highly porous, low‐density cellulose nanofiber (CNF) foams are prepared by a simple, environmentally friendly, time‐saving, and economical process. The prepared citric acid‐crosslinked CNF foams exhibit high mechanical and antioxidant properties, good thermal stability, and good sound‐absorbing properties for promising environment‐friendly sound‐absorbing and packaging applications.

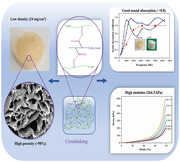

## 1 Introduction

The unique properties and advantages of the lightweight porous materials make them suitable for a wide range of applications, from sound absorption and thermal insulation to biomedicals. Different conventional synthetic and inorganic materials have been used to prepare these porous materials. For example, polyvinyl chloride, ethylene‐vinyl acetate, and ethylene propylene diene monomer (EPDM) foams are widely used as sound‐absorbing materials. Since they are petroleum‐based, environment‐friendly, and bio‐based materials, are receiving much attention from academia and industries as substitutes for conventional synthetic materials. Several bio‐based materials, such as cellulose, chitosan, starch, and alginate, have been used to prepare lightweight porous materials.^[^ ^1^ ^]^ They are environment‐friendly, renewable, abundant, and low cost.

Cellulose, the most abundant biopolymer in nature, is a linear homopolymer of β(1‐4)‐linked glucose residues and the major polymer of plant cell walls. With many advantages of lightweight, non‐toxicity, non‐abrasiveness, and renewability, it offers good mechanical, physical, and thermal properties that make it the most promising candidate for the preparation of lightweight porous materials.^[^ ^1^, ^2^, ^3^, ^4^, ^5^, ^6^ ^]^ Cellulose can be isolated from natural resources in cellulose nanocrystal (CNC) and cellulose nanofiber (CNF), called nanocellulose. Nanocellulose has many excellent properties: large specific surface area, low thermal expansion coefficient, optical transparency, and good reactivity. These characteristics make nanocellulose suitable for environment‐friendly and lightweight porous materials for various applications, including thermal insulation,^[^ ^7^, ^8^, ^9^ ^]^ flame retardancy,^[^ ^9^, ^10^, ^11^ ^]^ sound absorption,^[^ ^12^ ^]^ biomedical,^[^ ^13^, ^14^, ^15^ ^]^ and energy storage.^[^ ^16^ ^]^

Generally, CNF foams are prepared by a sol–gel method followed by drying to remove or replace the solvent with air or gas.^[^ ^6^ ^]^ During the gel formation, the sol–gel transition of nanofibers creates 3D networks via physical or chemical crosslinking. The final porous structure and material performance depend on this crosslinking method. Chemical crosslinking allows new covalent bonds between nanocellulose and reactive crosslinking agents to significantly improve the material's performance. Various crosslinking agents are used to prepare the CNF‐based porous materials, for example, maleic acid,^[^ ^17^ ^]^ acrylic acid,^[^ ^18^ ^]^ 1,2,3,4‐Butanetetracarboxylic acid (BTCA),^[^ ^11^ ^]^ silanes,^[^ ^16^, ^19^, ^20^, ^21^ ^]^ trimethylolpropane‐tris‐(2‐methyl‐1‐aziridine) propionate (TMPTAP), polyethyleneimine (PEI),^[^ ^22^ ^]^ polyamide‐epichlorohydrin resin,^[^ ^23^ ^]^ and dialdehyde starch.^[^ ^24^ ^]^ Most of them are inorganic or synthetic, toxic, and expensive. The use of these toxic and expensive inorganic or synthetic crosslinking agents causes health and environmental hazards and limits the applications of these porous materials. Therefore, this study used citric acid (CA) as a bio‐based, environment‐friendly, low‐cost, non‐toxic crosslinking agent, termed as a green crosslinking agent.^[^ ^25^ ^]^

The most important part of the foam preparation is the drying method that controls the porous 3D structure of the foam. The main challenge is maintaining the original shape and porous foam structure after drying without deformation, collapse, or shrinkage. Freeze‐drying, lyophilization,^[^ ^11^ ^]^ supercritical drying,^[^ ^26^ ^]^ evaporation, and oven drying^[^ ^27^, ^28^ ^]^ are commonly used methods for drying CNF foams or porous materials. Both freeze‐drying and supercritical drying methods are high‐cost, high‐energy, and time‐consuming because they require expensive special equipment and a complex operation. Thus, they are limited to only lab‐scale production and unsuitable for large‐scale production.^[^ ^5^, ^6^ ^]^

On the other hand, evaporation or oven drying is a relatively easy, time‐ and energy‐saving method with simple equipment suitable for scalable production. However, during evaporation drying, the porous structure of the prepared foam can collapse. It is difficult to maintain the original structure by avoiding shrinkage due to the capillary forces.^[^ ^5^, ^6^ ^]^ Cervin et al. prepared a CNF foam by adding octylamine to make CNF better adsorbed on the gas–liquid interface and then dried it using an oven.^[^ ^28^ ^]^ Some researchers used solvent (water) exchange with low or non‐polar solvents to reduce the interfacial tension during the drying process and improve the porous structure. Toivonen et al. prepared a CNF aerogel using solvent exchanged with 2‐propanol and octane for 24 h, followed by ambient drying that showed only 60% porosity and a density of 60 mg cm^−3^.^[^ ^29^ ^]^ Li et al. prepared a CNF aerogel by solvent exchange of frozen CNF with 2‐propanol for 72 h followed by oven drying.^[^ ^30^ ^]^ A CNF xerogel was also prepared by different solvent exchanges (ethanol, acetone, hexane, and pentane) for 72 h, followed by evaporation drying, and the resultant xerogel showed porosity of only 71–76%.^[^ ^31^ ^]^ A CNF aerogel was prepared using glycidoxypropyltrimethoxysilane (GPTMS) and PEI for crosslinking of CNFs obtained by solvent exchange with acetone followed by oven drying. The resultant aerogel showed a density of 58.82 mg cm^−3^.^[^ ^24^ ^]^ Recently, a CNF/alginate/CaCO_3_ hybrid aerogel was also prepared by solvent exchange, followed by air drying developed by overnight freezing of hydrogel and then soaking them in the acetic acid solution for 1 h before the solvent exchange with acetone.^[^ ^32^ ^]^ However, all the previous methods require long‐time preparation or more complex procedures and equipment to produce CNF‐based porous materials.

Therefore, we report an easy, environment‐friendly, time‐saving, and simple process to produce lightweight, highly porous CNF foam using citric acid as a green crosslinking agent. The foam was prepared by simple mixing, freezing, solvent exchange, and oven drying. As no special equipment is required, this process can be applied for scalable production. Moreover, a freeze‐drying method was used for comparison. The morphology and chemical interaction of the prepared foam were analyzed by scanning electron microscope and Fourier transform infrared (FTIR). The foam's mechanical, thermal, antioxidant, and hydrophobic properties were also investigated. Finally, the sound‐absorbing properties of the prepared foams were tested using an impedance tube method.

## 2 Results and Discussion

### 2.1 Chemical Interaction

**Figure 1:**
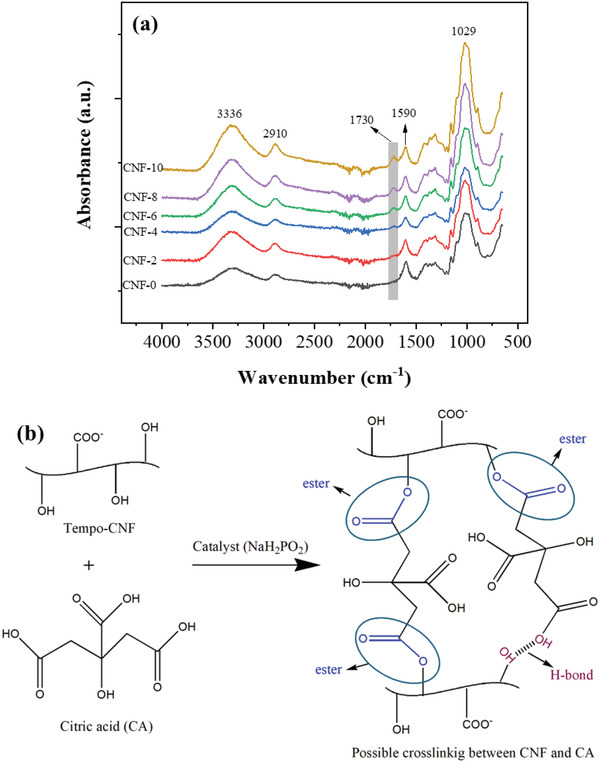
a) ATR‐FTIR spectra of the CNF foams and b) the crosslinking mechanism between CNF and CA.

The ATR‐FTIR spectra of the neat CNF foam (**Figure** **1**a) exhibit a broad peak at around 3336 cm^−1^, corresponding to the OH groups present on CNF and the adsorbed moisture on their surface. The peak at 2910 cm^−1^ corresponds to the CH stretching vibration. The absorption peak at 1590 cm^−1^ is due to the C=O stretching vibration of 2,2,6,6‐Tetramethylpiperidinyloxy‐CNF, and the 1029 cm^−1^ peak corresponds to the C—O stretching of the tempo‐CNF. Since the neat CNF foam was prepared without adding CA, only hydroxyl groups of CNF react with other hydroxyl groups of CNF through physical crosslinking via hydrogen bonding, showing a broad peak at around 3336 cm^−1^ (Figure 1a).

The spectra of all CA‐based CNF foams exhibit a new peak at 1730 cm^−1^. This new peak arises due to C=O stretching vibration that establishes the formation of the ester (—CO—O—) between OH groups of CNF and carboxylic groups of CA, confirming the chemical crosslinking between CNF and CA (Figure 1b). Hydrogen bonds may also be formed between CNF and CA. The degrees of crosslinking of the CNF‐0, CNF‐2, CNF‐4, CNF‐6, CNF‐8, and CNF‐10 were 98.88%, 98.76%, 98.84%, 98.96%, and 99.99%, respectively, indicating that most of the CA content was reacted with CNF. When CA is added to the CNF in the presence of a catalyst, anhydride is formed during the mixing and facilitates the formation of the esters in between OH groups of CNF and carboxyl acid groups of CA, developing an interconnected network structure through chemical crosslinking.^[^ ^25^ ^]^ Moreover, as increasing the CA content, the peak intensities of the 1730 and 1029 cm^−1^ increased, indicating the increased density of esters formation. With the increasing CA content, the carboxylic acid groups also increase to interact with more hydroxyl groups of the CNF and thus generate more ester bonds. Compared to the weak hydrogen bonding, the strong and stable chemical crosslinking via esterification is more important to maintain the shape and porous structure of CNF foams and thus plays a key role in the mechanical performance of the foams.

### 2.2 Foam Shrinkage, Density, and Porosity

**Figure 2:**
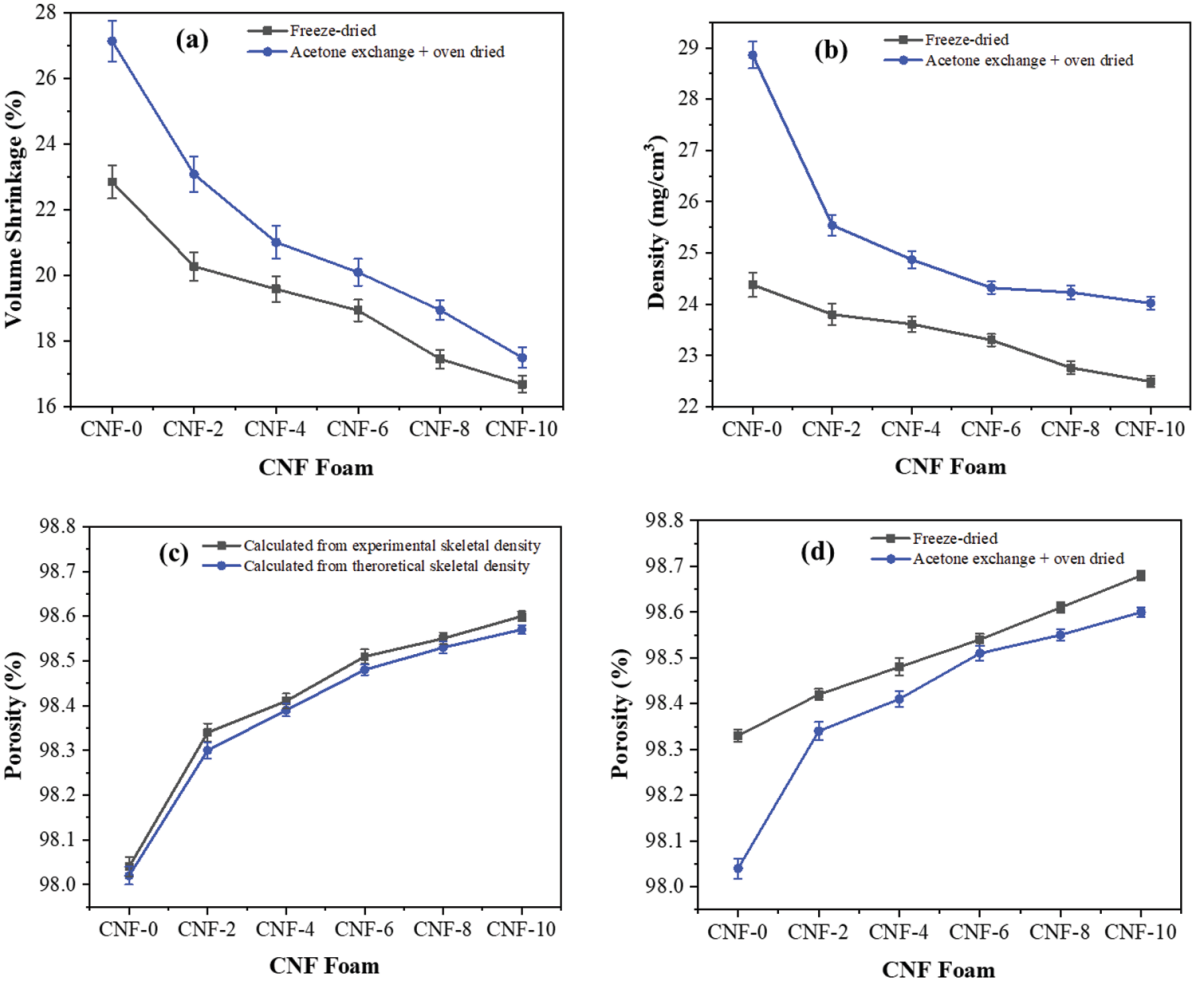
CNF foam parameters with CA content: a) volume shrinkage, b) density, and c,d) porosity.

The volume shrinkage of the CNF foams is presented in **Figure** **2**a. The samples CNF‐0, CNF‐2, CNF‐4, CNF‐6, CNF‐8, and CNF‐10 show a shrinkage of 27.14%, 23.08%, 21.01%, 20.09%, 18.94%, and 17.49%, respectively. The neat CNF foam shows slightly higher shrinkage, but the addition of CA in the CNF reduces the shrinkage and improves the foam structure indicating a maximum of 35.55% reduction in shrinkage for the CNF‐10 compared to the neat CNF foam. Note that the samples prepared by the current method show a slightly higher shrinkage than the freeze‐dried samples. However, the difference is decreased with increasing the CA content, and the foam with 10wt% CA shows almost similar shrinkage with a minor difference of 4.85%. The bulk density in Figure 2b indicates that the neat CNF foam density is 28.86 mg cm^−3^, whereas the CA‐crosslinked CNF foams show a density between 25.54 to 24.02 mg cm^−3^. The foam density decreased linearly with increasing the CA content, and the foam with 10wt% CA content showed the lowest density (24.02 mg cm^−3^). The CNF foams prepared in this study showed a much lower density than the previously reported CNF aerogel^[^ ^29^ ^]^ and GPTMS‐ and PEI‐crosslinked CNF aerogel^[^ ^24^ ^]^ prepared by solvent exchange followed by evaporation drying. Although the density of the neat CNF foam prepared by the current method shows slightly higher than the freeze‐dried samples, the CA‐crosslinked foams are not far from the freeze‐dried samples.

The porosity data of CNF foams calculated from the experimental and theoretical skeletal densities are shown in Figure 2c, and all the CNF foams show almost the same porosity value with a negligible difference of less than 0.04% when calculated from experimental and theoretical skeletal density. The neat CNF foam indicates a porosity of 98.04% that increased up to 98.60% as the CA content increased. The highest porosity for the CNF‐10 sample (98.60%) is much higher than the previously reported CNF aerogels prepared by solvent exchange followed by evaporation^[^ ^29^, ^31^ ^]^ and the freeze‐dried CNF foam prepared using cationic surfactants.^[^ ^33^ ^]^ Compared with freeze‐dried samples, the CA‐crosslinked CNF foams prepared by the current method show similar values (Figure 2d). During the water–acetone exchange process, the water was exchanged by lower surface tension acetone that reduces the capillary force experienced by the foam; finally, the 3D porous structure of the samples was preserved by preventing them from shrinkage during the drying. Therefore, the prepared foam shows low shrinkage, low density, and very high porosity.

Further, the reduced shrinkage, density, and improved porosity of the CA‐crosslinked CNF foams have resulted from chemical crosslinking between CNF and CA. The addition of CA to the CNF strengthens the pore cell walls of the foam and prevents them from collapsing during drying, creating a robust 3D network structure through chemical crosslinking, resulting in a homogeneous 3D cellular structure throughout the material which further improved with increasing CA content. So, the current processing method did not significantly influence the foam shrinkage, density, and porosity, and showed almost similar microstructural properties comparable to the samples prepared by the freeze‐drying method, which requires a long processing time and cost.

### 2.3 Morphology

**Figure 3:**
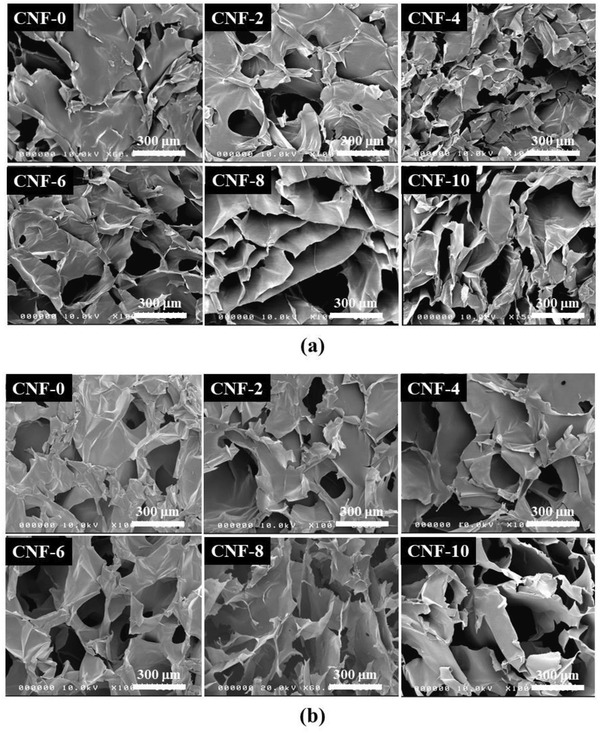
SEM images of CNF foams: a) acetone exchange and oven dried, b) freeze‐dried.

The SEM micrographs of the neat CNF foam (**Figure** **3**) show an irregular 3D porous network indicating a randomly oriented 2D sheet‐like structure created by pushing the CNFs into a 2D sheet‐like geometry during the ice crystals growth when freezing due to the weak hydrogen bonding. Therefore, the neat CNF foam does not maintain a proper 3D structure indicating large shrinkage and pore collapse during drying. With a small amount of CA (2 wt%), the foam showed a microstructure similar to the neat CNF foam but with larger interlayer spacing. Further increase of the CA content improves the pore size, shape, and connectivity with a good distribution of the interconnected pores having a well‐organized structure inter‐connected through solid and consistent cell walls. However, in the freeze‐dried samples (Figure 3b), the foam samples show slightly better microstructure with larger interlayer spacing than those prepared by the current method. Relative to the neat CNF foam, the well‐connected pores through strong cell walls resulted from chemical crosslinking by strong ester bonds that enhance the porosity, resulting in low density and highly porous CNF foam.

### 2.4 Mechanical Properties

**Figure 4:**
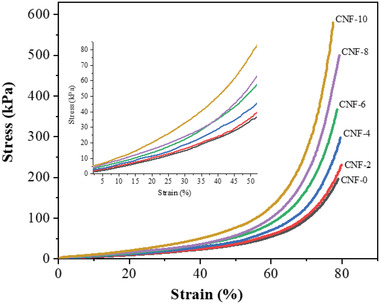
Representative compressive stress–strain curves of acetone exchange + over dried CNF foams.

**Figure** **4** exhibits the CNF foams’ representative compressive stress versus strain curves prepared by acetone exchange + over‐dried, and their compressive properties are recorded in **Table** **1**. The neat CNF foam is deformed at a low compressive load showing a compressive strength of 4.81 kPa at 10% strain and 140.29 kPa at 75% strain. In the neat CNF foam, the CNFs are joined together by only mechanical entanglement and physical crosslinking by hydrogen bonding; therefore, the foam shows a low compressive strength over the entire strain range.

**Table 1:** Mechanical properties of the CNF foams

Sample	Compressive strength at 10% strain [kPa]		Compressive strength at 50% strain [kPa]		Compressive strength at 75% strain [kPa]		Compressive modulus [kPa]	
	Acetone exchange + oven dried	Freeze‐dried	Acetone exchange + oven dried	Freeze‐dried	Acetone exchange + oven dried	Freeze‐dried	Acetone exchange + oven dried	Freeze‐dried
CNF‐0	4.81 (±0.21)	5.21 (±0.24)	35.39 (±1.92)	36.68 (±1.55)	140.29 (±5.62)	145.21 (±4.99)	76.67 (±4.97)	81.93 (±4.99)
CNF‐2	5.69 (±0.31)	5.88 (±0.29)	37.52 (±1.97)	38.77 (±2.21)	158.83 (±5.59)	163.29 (±4.91)	126.42 (±6.58)	130.84 (±6.10)
CNF‐4	6.97 (±0.24)	7.20 (±0.39)	43.44 (±2.23)	43.96 (±9.89)	202.86 (±7.26)	209.18 (±6.89)	146.25 (±7.29)	151.55 (±6.28)
CNF‐6	8.09 (±0.38)	8.31 (±0.36)	53.95 (±2.76)	54.97 (±2.71)	280.60 (±7.91)	289.63 (±9.21)	173.34 (±9.24)	177.18 (±8.63)
CNF‐8	9.31 (±0.44)	9.51 (±0.38)	59.02 (±3.04)	60.05 (±2.12)	332.64 (±9.43)	339.51 (8.68)	206.50 (±8.66)	210.41 (±7.85)
CNF‐10	10.82 (±0.45)	10.98 (±0.41)	76.07 (±3.77)	76.92 (±3.19)	440.67 (±11.41)	450.08 (±13.08)	264.54 (±9.83)	271.45 (±9.91)

The compression strength of CA‐crosslinked CNF foams is higher than that of the neat CNF foam (Table 1) and continues to increase with the CA content over the entire strain range. Compared to the neat CNF foam, the compression strength (at 10% strain) of the CNF‐2, CNF‐4, CNF‐6, CNF‐8, and CNF‐10 are increased by 13.22%, 44.60%, 100.01%, 137.11%, and 214.11%, respectively, and at 75% strain are 18.30%, 44.91%, 68.19%, 93.56%, and 124.95%, respectively. Similarly, the compression modulus of the CNF‐2, CNF‐4, CNF‐6, CNF‐8, and CNF‐10 increased by 64.89%, 90.75%, 126.08%, 169.33%, and 245.04%, respectively. Compared to the Freeze‐dried samples (Table 1), although the neat CNF foam prepared by the current method shows a slightly lower compression strength over the entire strain range (8.31%, 3.64%, and 3.50% lower strength at 10%, 50%, and 75% strain, respectively) and a lower compression modulus (6.86% lower modulus). However, in the case of CA‐crosslinked foams, this difference is reduced with the increasing CA content, and foam with 10wt% CA shows almost similar values with a minor difference of only 1.47%, 1.11%, and 2.13% in the compression strength at 10%, 50%, and 75% strain, respectively, and a minor difference of only 2.76% in the compression modulus. The compression modulus of the CNF foams prepared by the current study was much higher than the reported freeze‐dried CNF foam prepared using cationic surfactants.^[^ ^33^ ^]^ The CA‐crosslinked foams’ much higher compression properties could be attributed to the chemical crosslinking via strong and stable ester bonds that lead to a condensed 3D cellular network structure (as discussed in Figure 1b), which contributes to improved strength and modulus. Further, the ester formation density increases with the CA content, indicating more crosslinking resulting in improved mechanical properties.

### 2.5 Thermo‐Gravimetric Study

**Figure 5:**
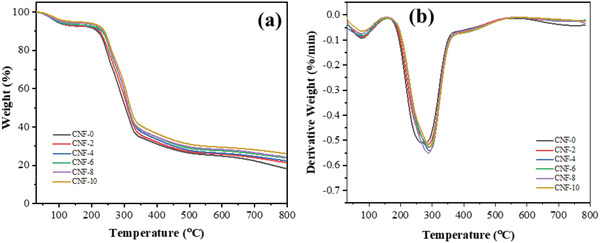
a) TG and b) DTG curves of CNF foams.

**Figure** **5** shows the TG and DTG thermograms of the CNF foams. Being hydrophilic, CNF contains moisture that gets released between 50 to 100 °C, with all samples recording a weight loss of ≈5%, indicating a DTG peak at around 78 °C (Figure 5b). The degradation of the neat CNF foam commences at 215 °C, then achieves a maximum degradation at 272 °C and finally approaches the end at 800 °C, leaving 18.33% char residual mass. The degradation of CNF‐2, CNF‐4, CNF‐6, CNF‐8, and CNF‐10 samples commences at 221, 228, 231, 235, and 237 °C. They achieve the maximum degradation at 286, 291, 291, 290, and 293 °C, leaving the char residue of 21.52%, 22.12%, 23.64%, 24.16%, and 25.89%. It is observed that the initial decomposition temperatures of CA‐crosslinked CNF foams are higher than those of the neat CNF foam and that further increases with the CA amount in them. The initial decomposition temperature of CNF foam prepared in this study is higher than that of the previously reported nanocellulose aerogel prepared with or without a phosphorus‐containing flame retardant modifying agent^[^ ^34^ ^]^ and is also much higher than that of the polyurethane foam that showed the initial decomposition temperatures at 210^[^ ^35^ ^]^ and 212 °C.^[^ ^36^ ^]^ In addition, all the CA‐crosslinked CNF foams show higher char residues than the neat CNF foam at 800 °C. It might be due to the chemical crosslinking via strong covalent ester bonds that lead to better thermal stability and generate high char residues.

### 2.6 Antioxidant Activity

**Figure 6:**
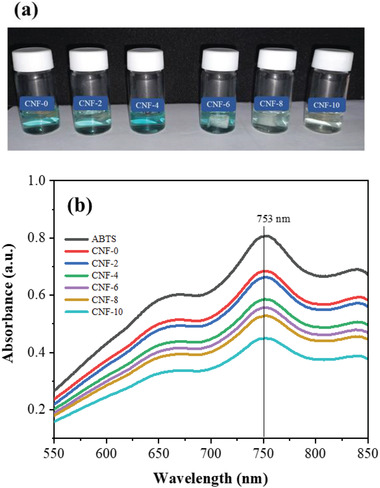
a) Color changes in the CNF foam samples containing ABTS solution, and b) their UV spectra after keeping them in the darkroom for 40 min.

The antioxidant activity of the prepared CNF forms was measured using the discolored radicals of ABTS under UV light measurement at 734 nm. The dilution of the ABTS solution with ethanol shifts the maximal absorption peak from 734 to 753 nm due to the solution effect.^[^ ^37^, ^38^ ^]^ **Figure** **6**a shows the dis‐coloration of CNF foams containing ABTS solution after keeping them in the darkroom for 40 min. The UV curves in Figure 6b calculate the antioxidant activity. The neat CNF foam shows an antioxidant activity of 14.50%, whereas the CNF‐2, CNF‐4, CNF‐6, CNF‐8, and CNF‐10, exhibit 17.38%, 27%, 30.63%, 34.35%, and 43.75%, where CNF‐10 showed the highest value. Although the CNF has low antioxidant activity, adding CA increased the foams’ antioxidant activity, indicating three times higher antioxidant activity of the CNF‐10 sample than the neat CNF foam. The antioxidant activity of the CA‐crosslinked CNF foam is higher than the reported chitosan nanofiber‐based CNF composite prepared with 10wt% chitosan nanofiber^[^ ^39^ ^]^ and is comparable with the reported tannic acid crosslinked chitosan nanocomposite film.^[^ ^40^ ^]^ CA is a natural antioxidant agent that can improve the antioxidant activity of the material.^[^ ^41^ ^]^ A similar enhancement in antioxidant activity with adding CA was also reported for the starch/cellulose film.^[^ ^42^ ^]^

### 2.7 Hydrophobicity

**Figure 7:**
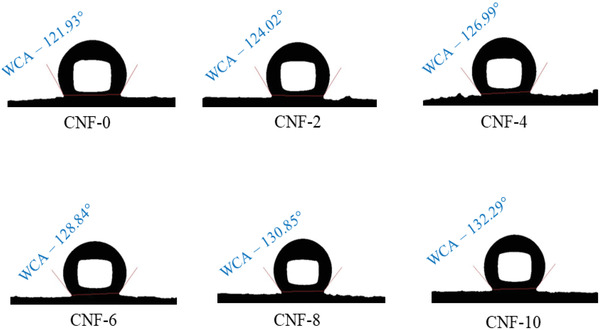
The water contact angle of ODA/VTMS treated CNF foams.

Hydrophobicity of foams is necessary for various applications. Thus, a water‐based ODA/VTMS solution was used for the hydrophobic treatment of CNF foams. When the water‐insoluble ODA was heated above its melting temperature, that is, above 55 °C, the ODA molecules were dispersed in the water, and the addition of the acetic acid into this dispersion formed ODA molecules in a petal‐like structure in the aqueous dispersion.^[^ ^43^, ^44^ ^]^ When VTMS, which has a very low hydrolysis rate and is slightly soluble in water, was added to the aqueous ODA dispersion, its molecules were easily adsorbed on ODA. The CNF is highly hydrophilic, having a reported WCA of around 40°. The ODA/VTMS solution treatment of the neat CNF foam made it hydrophobic, indicating a WCA of 121.93° (**Figure** **7**). When the neat CNF foam was treated with the ODA/VTMS solution, the VTMS formed an O—Si covalent linkage with the CNF and the amine groups of ODA formed hydrogen bonds with CNF. Also, the long hydrophobic alkyl chain of VTMS and ODA interact via van der Waals interactions.^[^ ^43^, ^44^ ^]^ VTMS works as a hydrophobic constituent to treat CNF foams by supporting aggregation via polymerization. Its addition to the ODA solution introduces the larger aggregation on the foam surface. The ODA/VTMS treated CNF‐2, CNF‐4, CNF‐6, CNF‐8, and CNF‐10 samples show WCAs of 124.02°, 126.99°, 128.84°, 130.85°, and 132.29° (Figure 7). All the treated CA‐crosslinked CNF foams increased their WCA by increasing the CA content. The WCA of the treated CNF foams by the current method is much higher than the previously reported TiO_2_ coated nanocellulose aerogel^[^ ^45^ ^]^ and is similar to that of the CNF aerogel treated with hexadecyltrimethoxysilane (HDTMS) by a chemical vapor deposition method.^[^ ^46^ ^]^ The addition of CA in CNF leads to crosslinking via ester bonds between them, where most of the OH groups of CNF are already linked with the CA and are not available to react with water molecules on a foam surface. Therefore, adding CA reduces the free OH on the foam surface and increases the WCA resulting in CA‐crosslinked ODA/VTMS treated CNF foams showing higher hydrophobicity than the neat CNF foam.

### 2.8 Sound‐Absorbing Behavior

**Figure 8:**
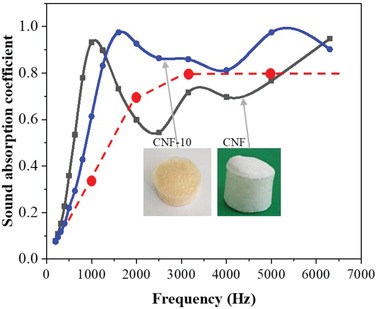
Sound‐absorbing behaviors of CNF foams.

The sound absorption coefficient of the CA‐crosslinked CNF foam, CNF‐10, was measured using the impedance tube method, and **Figure** **8** shows the result. The red dotted line indicates the required sound absorption coefficient of polyurethane foam for automobile applications. The neat CNF foam's coefficient is shown for comparison. The neat CNF foam represents a lower sound absorption coefficient than the required value over 2000 Hz, but the CNF‐10 shows a higher value. The high sound absorption coefficient of CNF‐10 might be associated with its high porosity. In conclusion, the CA‐crosslinked CNF foam can be an environment‐friendly sound‐absorbing material.

## 3 Conclusion

This study reported a simple, environmentally friendly, time‐saving, and simple process to prepare the CNF foams that may be applied for large‐scale production. The foams were prepared using citric acid as a green crosslinking agent that introduced chemical crosslinking in the foam via strong ester bonds, which FTIR confirmed. The prepared foams were highly porous (more than 98%), having a low density of 24.02 mg cm^−3^ with a minor shrinkage, comparable to the freeze‐dried foam. The SEM images of the foams indicate a well‐maintained porous 3D cellular structure due to the chemical crosslinking that improved compressive strength and modulus up to 214% and 245%, respectively, and the thermal stability of the foam was also increased. The antioxidant activity of the foam increased up to three times with the addition of citric acid.

Further, the hydrophobic treatment was also applied to the CA‐crosslinked CNF foams, and the hydrophobicity of the foams was also improved with the increasing citric acid content. Finally, the sound‐absorbing performance of the prepared foam was tested, and it showed higher sound absorption coefficient values than the required ones. Low density, high porosity, high mechanical properties, high antioxidant property, good thermal stability, and sound‐absorbing behaviors of the CA‐crosslinked CNF foam are promising for environment‐friendly sound‐absorbing and packaging applications.

## 4 Experimental Section

### Materials

Tempo‐oxidized CNF as an aqueous suspension with 2.05wt% concentration was obtained from Moorim Paper Co. Ltd., South Korea. Citric acid (CA) (99%), sodium hypophosphite (SHP) (99%), 2,2′‐azino‐bis(3‐ethylbenzothiazoline‐6‐sulfonic acid) (ABTS) solution, potassium persulfate (99%), octadecylamine (ODA) (99%), and vinyltrimethoxysilane (VTMS) (98%) were obtained from Sigma Aldrich. Acetic acid was obtained from Wako Pure Chemical Industries Ltd., South Korea. All the chemicals were used without purification, and de‐ionized (DI) water was used throughout the experiment.

### Foam Preparation

**Figure 9:**
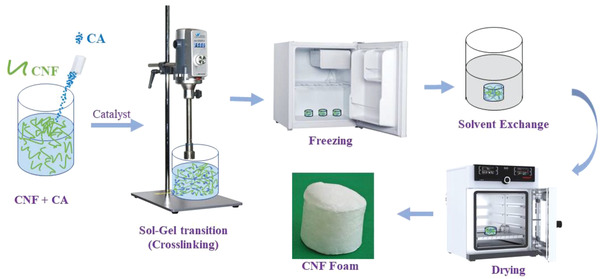
Systematic presentation of CNF foam preparation.

CNF foam was prepared by the sol–gel method using chemical crosslinking. The scheme used for the foam preparation is shown in **Figure** **9**. The CNF concentration was diluted to 1.5wt% to prepare CNF foams. Different amounts of CA were added to the CNF, and subsequently, SHP was added as a catalyst for the direct crosslinking of CA with CNF. The SHP content was always half of the CA content. The CNF, CA, and SHP were mixed using a homogenizer (IKA T25 D, Germany) at 6000 rpm for 20 min followed by 3000 rpm for 10 min to obtain a homogeneous CNF gel‐like gel solution. The above solution was poured into a bottomless cylindrical plastic mold, placed on a stainless‐steel plate and frozen in an ordinary freezer at −18 °C for 3 h. The frozen samples were soaked in acetone for 2 h to water–acetone exchange (acetone was changed after 1 h). Finally, the acetone‐infused samples were dried using a vacuum oven at 100 °C for 3 h and designated CNF‐0, CNF‐2, CNF‐4, CNF‐6, CNF‐8, and CNF‐10. The number denotes the wt% of CA relative to the total dry weight of CNF. The CNF foams were prepared using a freeze‐dryer (Eyela FDU‐2200, Japan) at −85 °C for 24 h.

### Chemical Interactions by FTIR

The ATR‐FTIR spectra of each material were recorded by FTIR Spectrometer (Agilent Technologies Cary 630 FTIR, South Korea) in the 4000 to 500 cm^−1^ range using ATR mode. Before the analysis, the samples were dried completely using an oven at 70 °C for 5 h to remove any present moisture. The degree of crosslinking was calculated by soaking the samples in acetone for 24 h and drying them at 80 °C for 12 h. The uncrosslinked fraction was supposed to be completely dissolved in acetone. The percentage degree of crosslinking was calculated by dividing the remained insoluble weight (*W*_2_) after soaking in acetone by the initial weight (*W*_1_) of the sample and multiplying by 100 [(*W*_2_/*W*_1_) × 100].

### Foam Shrinkage, Density, and Porosity

The volume shrinkage was calculated by measuring their dimensions (width and height) using a digital caliper (Mitutoyo 293–801, Japan) with 1 µm resolution. The percentage volume shrinkage was calculated using the following equation.

(1) $$\begin{array}{c} \text{Volume shrinkage}(\%)=\left(\frac{V_{m}-V_{s}}{V_{m}}\right)\;\times \;100 \end{array}$$

where, *V*_m_ and *V*_s_ are the volumes of the mold and sample after drying, respectively.

The bulk density was calculated by dividing their weight by volume, measured by a digital caliper and digital balance (A&D GH‐200, Japan). The porosity was calculated from the following equation.

(2) $$\begin{array}{c} \text{Porosity}\left(\%\right)=\left(\frac{\rho_{\text{sc}}-\rho_{\text{bulk}}}{\rho_{\text{sc}}}\right)\;\times \;100 \end{array}$$

where ρ_bulk_ is the bulk density, and ρ_sc_ is the skeletal density of the material. The skeletal density was measured by gas pycnometer (BELPYCNO, Microtac MRB) by gas displacement method using helium gas. Theoretically, the skeletal density was calculated from the weighted average of the densities of the components, that is, the density of CNF = 1.460 gm cm^−3^,^[^ ^9^ ^]^ the density of CA = 1.665 gm cm^−3^, and the density of SHP = 1.388 gm cm^−3^.

### Morphology

The SEM pictures were taken to investigate the morphology and microstructure of CNF foams by FE‐SEM (S‐4000, Hitachi, Japan) using an accelerating voltage of 10 kV. The platinum coating was applied to the samples before the examination using an ion sputter (Emitech, K575X, UK).

### Compression Test

The compression test was conducted at ambient conditions (25 °C) following the ISO 844:2014 on a universal testing machine operated at a 1 mm min^−1^ crosshead speed. The cylindrical samples of 10 mm in height were used for the testing, and the average results of five samples were recorded.

### Thermo‐Gravimetric Analysis

Thermo‐gravimetric analysis was performed by the TGA (TG 209 F3 Tarsus, NETZSCH, Germany) using an average 7 mg sample of a material heated at 10 °C min^−1^ in an inert nitrogen atmosphere from room temperature up to 800 °C.

### Antioxidant Activity Test

The antioxidant activity was measured by assessing the free radical scavenging activity. The ABTS radical scavenging method was used for the antioxidant test.^[^ ^40^ ^]^ 7 mm ABTS solution was mixed with 2.45 mm potassium persulfate solution in the ratio of 2:1 and was kept in the darkroom for 12–16 h. Then the 734 nm absorption peak was adjusted at 0.8 wavelengths using ethanol. 30 mg of each material in 3 mL of the above ABTS solution was measured after being in a dark room for 40 min. The antioxidant activity was calculated using Equation (3) and expressed in AO% per 100 mg:

(3) $$\begin{array}{c} \text{AO}\%=\left(\frac{A_{0}-A_{S}}{A_{0}}\right)\;\times \;100 \end{array}$$

where *A*_0_ and *A*_S_ are the absorptions of the control ABTS solution and the sample containing ABTS solution in a steady state.

### Hydrophobic Treatment and Hydrophobicity

A simple water‐based method was used for the hydrophobic treatment. For this, ODA and VTMS were mixed in a 1:3 molar ratio. First, 27 mg ODA was dispersed in 50 ml of DI water using acetic acid (pH adjusting at 7.0) to obtain a homogeneous solution, and then 44 mg VTMS was added to the above solution. The mixture was stirred at 300 rpm, 60 °C to disperse VTMS completely into the solution, and a clear and homogeneous mixture was obtained after 1 h. The foams were soaked into the above solution for 15 min at 60 °C, followed by drying in an oven at 100 °C for 5 h. The treated foams’ water contact angle (WCA) was measured using a contact angle measuring instrument (Surfacetech, GSA, South Korea) by placing a water droplet on the sample's surface.

### Sound Absorption Coefficient

The sound absorption coefficient represents the sound‐absorbing performances of a material. An impedance tube method was used to measure the sound absorption coefficient, *α_n_* when a normal incidence is applied.^[^ ^47^ ^]^

(4) $$\begin{array}{c} \alpha_{n}=1-{\left(\frac{\log_{10}^{-1}\left(L/20\right)-1}{\log_{10}^{-1}\left(L/20\right)+1}\right)}^{2} \end{array}$$

Here, *L* is the difference in decibels between the maximum and minimum sound pressure levels in the standing wave pattern in the tube.

## Conflict of Interest

The authors declare no conflict of interest.

## Data Availability

The data that support the findings of this study are available from the corresponding author upon reasonable request.
